# Imaging of malignancies of the biliary tract- an update

**DOI:** 10.1186/1470-7330-14-14

**Published:** 2014-04-22

**Authors:** Tiffany Priyanthi Hennedige, Wee Thong Neo, Sudhakar Kundapur Venkatesh

**Affiliations:** 1 Department of Diagnostic Imaging, National University Health System, 5 Lower Kent Ridge Road, Singapore 119074, Singapore; 2 Department of Radiology, Mayo Clinic College of Medicine, 200 First Street SW, Rochester 55905, USA

**Keywords:** Biliary tract, Malignancies, CT, MRI, Ultrasound, Cholangiocarcinoma, Gall bladder carcinoma, Ampullary carcinoma

## Abstract

Malignancies of the biliary tract include cholangiocarcinoma, gallbladder cancers and carcinoma of the ampulla of Vater. Biliary tract adenocarcinomas are the second most common primary hepatobiliary cancer. Due to their slow growing nature, non-specific and late symptomatology, these malignancies are often diagnosed in advanced stages with poor prognosis. Apart from incidental discovery of gall bladder carcinoma upon cholecystectomy, early stage biliary tract cancers are now detected with computed tomography (CT) and magnetic resonance imaging (MRI) with magnetic resonance cholangiopancreatography (MRCP). Accurate characterization and staging of these indolent cancers will determine outcome as majority of the patients’ are inoperable at the time of presentation. Ultrasound is useful for initial evaluation of the biliary tract and gallbladder masses and in determining the next suitable modality for further evaluation. Multimodality imaging plays an integral role in the management of the biliary tract malignancies. The imaging techniques most useful are MRI with MRCP, endoscopic retrograde cholangiopancreatography (ERCP), endoscopic ultrasound (EUS) and positron emission tomography (PET). In this review we will discuss epidemiology and the role of imaging in detection, characterization and management of the biliary tract malignancies under the three broad categories of cholangiocarcinomas (intra- and extrahepatic), gallbladder cancers and ampullary carcinomas.

## Introduction

Biliary tract cancer is the second most common primary hepatobiliary malignancy after hepatocellular carcinoma. Malignancies may occur along any part of the biliary tract from the ampulla of Vater to the smallest intrahepatic ductules and the gallbladder [1]. The entire biliary tree, including the gallbladder is lined with a simple columnar epithelium and malignant transformation of this epithelium gives rise to predominantly adenocarcinomas [2]. The pathogenesis of biliary tract and gallbladder carcinoma is thought to be secondary to an evolutionary sequence from metaplasia to dysplasia to carcinoma. Metaplasia usually occurs in the setting of inflammation and chronic injury. Dysplasia of the biliary tract, is considered as pre-invasive biliary neoplasia and can occur in up to 40% to 60% of patients with invasive carcinoma, one third of patients with sclerosing cholangitis, and found incidentally in 1 to 3.5% of cholecystectomy specimens [3,4].

**Figure 1:**
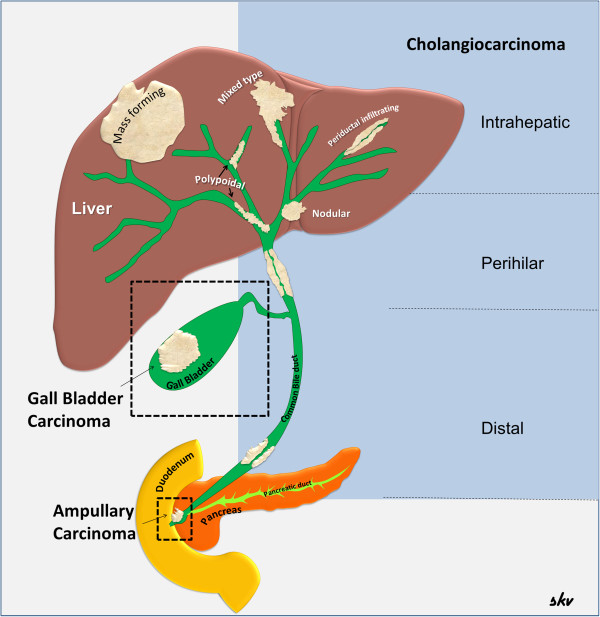
**Malignancies of the biliary tract.** Diagram showing the biliary tract and the various malignancies arising from the tract. The cholangiocarcinomas are illustrated according to current classification into anatomical and morphological subtypes.

Classically, the cancers of the biliary tract were separated into three categories: (i) cancer of the intrahepatic biliary tract, (ii) cancer of the gallbladder and extrahepatic bile ducts, and (iii) cancer of the ampulla of Vater [5]. The term cholangiocarcinoma was initially used to refer only to the primary tumors of the intrahepatic bile ducts and is now extended to include intrahepatic, perihilar, and distal extrahepatic tumors of the bile ducts [6] (Figure 1). Gallbladder cancer is defined as cancer arising from the gallbladder and the cystic duct. Ampullary cancers are rare and have better prognosis than cancers of the distal bile duct. Cancers arising from the distal common bile duct immediately adjacent to the ampulla of Vater tend to behave clinically similar to the cancers of the ampulla of Vater, head of pancreas and the duodenal bulb and therefore often considered under the broad category of periampullary carcinomas [7].

Tumors of the biliary tract may cause signs and symptoms from abdominal pain or jaundice. Biliary tract malignancies are slow growing and often diagnosed late with poor prognosis due to the non-specific and late stage symptomatology. The symptoms may occur early if the tumor is located in the common hepatic duct, the common bile duct or the ampulla of Vater. Apart from incidental discovery upon cholecystectomy, early stage biliary tumors are now increasingly diagnosed with computed tomography (CT) and magnetic resonance imaging (MRI).

Clinical history, geographic background, risk factors, patient’s age and gender are often crucial for arriving at diagnosis of these tumors. Accurate characterization and staging of the malignancies will determine resectability and impact on subsequent management. Multimodality imaging plays a deep and integral role in the management of malignancies of the biliary tract. The techniques most useful are MRI with magnetic resonance cholangiopancreatography (MRCP), positron emission tomography (PET), endoscopic retrograde cholangiopancreatography (ERCP) and endoscopic ultrasound (EUS). In this review, we will discuss the role of imaging in detection, characterization and management of the biliary tract malignancies under the three broad categories of cholangiocarcinomas (intra- and extrahepatic), gallbladder cancers and ampullary carcinoma.

## Review

### Cholangiocarcinoma (CCA)

#### Epidemiology

CCA is the most common biliary malignancy [8] but is a rare tumor that comprises less than 2% of all cancers [9]. It arises from bile duct epithelium and is the second most common primary hepatobiliary cancer after hepatocellular carcinoma (HCC). Among gastrointestinal tumors, it is the most difficult to detect and diagnose and has a poor prognosis with a 5-year survival rate of less than 5% [10]. CCA is uncommon in many parts of the world such as Europe and the USA with incidence estimates ranging from 0.8 to 2 per 100,000 [11]. However, there is a distinct geographic variation in their incidence, with the highest prevalence in Southeast Asia [12]. This reflects regional differences in risk factors and epidemiology. Risk increases with age, with peak prevalence occurring in the 7^th^ decade and has a slight male predilection [13].

At histopathology, 95% of cases are adenocarcinomas [14]. The histological grade of tumors can range from well differentiated to undifferentiated types. Other tumor types include squamous cell carcinoma, adenosquamous carcinoma and small cell carcinoma.

Generally CCAs occur sporadically with no identifiable risk factors. Chronic biliary inflammation is a common risk factor of CCA [15]. Well-documented risk factors include primary sclerosing cholangitis (PSC), choledochal cyst, familial polyposis, hepatolithiasis, congenital hepatic fibrosis, clonorchiasis and a history of exposure to thorotrast [13]. PSC is the best-known risk factor with a lifetime prevalence of CCA ranging from 5-15% and an overall risk of 0.5%-1.5% per year [16]. Nearly 50% of patients are diagnosed with CCA within a year of diagnosis of PSC [16]. Patients with cystic bile duct disorders like Caroli’s disease and choledochal cyst have high lifetime incidence of CCA ranging from 6% to 30% [17]. They also tend to have CCA diagnosed at a younger age than the general population [12,17] and many still develop CCA after resection of choledochal cysts. A higher prevalence of positive anti–hepatitis C virus antibody has also been reported to be associated with CCA and the risk of developing CCA in cirrhotic patients is ten-fold higher than the general population [12,18]. In Southeast Asia where the tumor is relatively more common, the associated risk factors are liver flukes (*Opisthorchis viverini* and *Clonorchis sinensis*) and chronic typhoid carriers with the latter carrying a six-fold increased risk of all hepatobiliary malignancies [14]. In addition, a genetic predisposition is suggested with a mutation in the p53 tumor suppressor and k-ras genes seen in intrahepatic and extrahepatic CCA respectively [19]. Lifestyle factors including heavy alcohol use, diabetes and obesity are also more prevalent in patients with CCA [20]. A strong association exists between Thorotrast, a radiologic contrast agent used before 1960, and the development of cholangiocarcinoma several years after exposure [12].

#### CCA classification and morphology

CCA are classified based on their anatomic location as intrahepatic CCA (iCCA), perihilar CCA (pCCA) and distal (dCCA) subtypes [6]. These different types are regarded as distinct entities from a treatment point of view [8]. The intrahepatic type, iCCA accounts for 10%, pCCA for 25-50% and dCCA for 40-65% of all CCA [21]. Some authors have also divided extrahepatic CCA into upper third (hilar), middle third and lower third [22]. CCA can also be classified on the basis of their macroscopic growth pattern into three types: (1) mass-forming exophytic type, which typically appears as a hepatic parenchymal mass; (2) periductal infiltrative type, of which tumor growth progresses along the bile duct longitudinally. This often results in upstream biliary tree dilatation and (3) intraductal polypoid type that proliferates focally within the lumen of the diseased bile duct [22]. Intrahepatic CCA are often of the mass-forming exophytic type, whereas extrahepatic variants mostly infiltrate longitudinally along the bile ducts [9]. The intraductal polypoid type is rare and can manifest in any of the subtypes. CCA can also exhibit a combination of growth patterns, and this is more frequently seen with intrahepatic tumors.

These gross growth-type morphologic characteristics enable interpretation of imaging features and assist in the differential diagnosis. More importantly, they can predict tumor dissemination and prognosis, which aids subsequent management including planning of surgical approach. More than 90% of CCA are well to moderately differentiated adenocarcinomas with desmoplastic reaction and early perineural invasion [23]. Malignancies associated with cystic anomalies of the bile duct or bile duct stones may be adenosquamous or squamous carcinomas.

#### Diagnosis of CCA

The diagnosis of CCA can be difficult due to silent growth and non-specific symptoms. Diagnosis requires a high degree of clinical suspicion in the appropriate setting of clinical presentation, laboratory, endoscopic and imaging findings. Patients with known risk factors need aggressive diagnostic work-up to confirm the diagnosis of CCA. Tumor markers like carbohydrate antigen 19–9 (CA19-9) can be raised, particularly with iCCA type [8]. Cut-off value of 129U/mL or greater detects iCCA in patients with PSC with a sensitivity and specificity of 79% and 98% respectively [24]. The primary role of imaging in CCA is to characterize the primary tumor, establish the presence or absence of satellite nodules or distant metastases, and identify the tumor’s relationship to the hepatic veins, inferior vena cava, the hepatic inflow pedicles, and the biliary tree. CT may be useful for volumetric assessment of potential liver remnants if patients are considered for surgical resection. Imaging techniques should also aim to identify extrahepatic lymph node disease as well as distant metastatic disease such as pulmonary or peritoneal metastases. Typically, patients with CCA are assessed with a CT of the chest, abdomen, and pelvis. An MRI may alternatively be used to stage the tumor.

### Intrahepatic CCA (iCCA)

**Figure 2:**
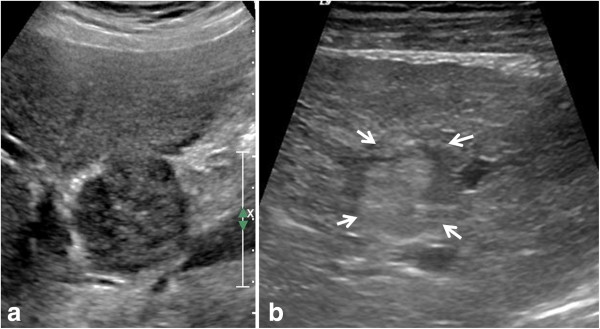
**Examples of intrahepatic cholangiocarcinoma (iCCA) on ultrasound in two different patients.** Mass forming iCCA may present as a well-defined hypoechoic mass (arrow, **a**) or as an ill-defined heterogeneous isoechoic mass (arrowheads, **b**).

**Figure 3:**
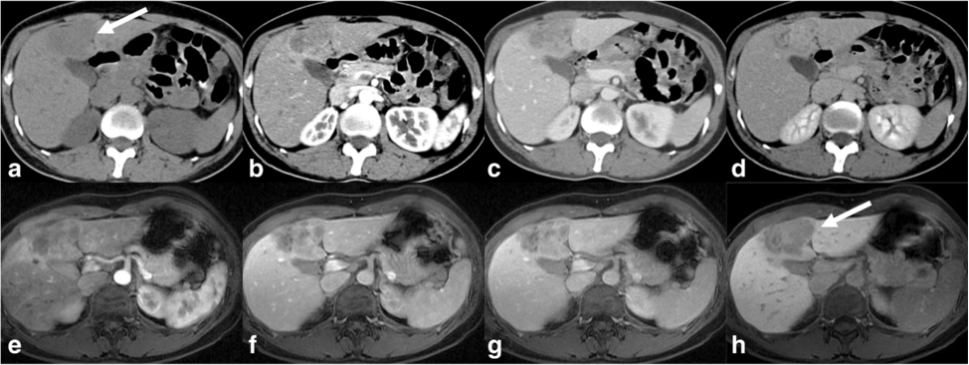
**CT and MRI appearances of mass forming type of iCCA.** Top row: non contrast enhanced **(a)**, post contrast enhanced arterial phase **(b)**, portal venous phase **(c)** and delayed phase **(d)** CT images. Bottom row: post contrast enhanced MRI images in arterial phase **(e)**, portal venous phase **(f)**, delayed phase **(g)**, and 20-minute delay post Gd-EOB-DTPA image **(h)**. The mass is iso- to hypodense to liver and shows similar contrast enhancement characteristics on both CT and MRI with peripheral rim like arterial phase enhancement with centripetal enhancement in the portal venous and delayed phases without any washout. In the hepatobiliary phase there is no uptake of Gd-EOB-DTPA by the mass suggesting a non-hepatocellular tumor.

**Figure 4:**
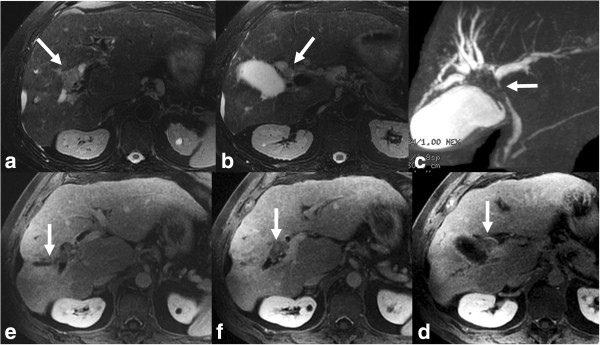
**Polypoid type iCCA.** Axial T2 sections **(a, b)** and MRCP **(c)** demonstrating an isointense filling defect (white arrow) in the right hepatic duct and extending into the common hepatic duct with dilation of intrahepatic ducts. Post contrast enhanced T1-weighted images **(d-f)** shows the mildly enhancing filling defect representing intraductal papillary neoplasm which extended from just under hepatic capsule filling right hepatic ducts to 3 cm below the confluence of right and left hepatic ducts.

**Figure 5:**
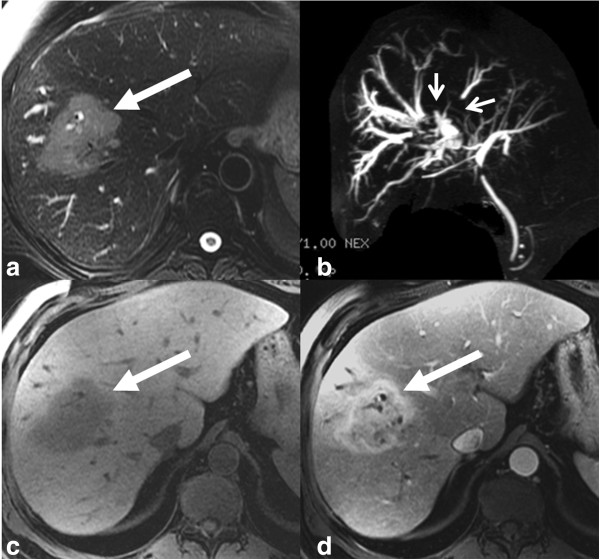
**Mixed type iCCA.** T2-weighted axial **(a)**, MRCP **(b)**, T1-weighted axial **(c)** and post contrast T1-weighted axial **(d)** images demonstrating a predominantly periductal thickening (stricturing iCCA) and also mass forming (arrow) in the right lobe liver. Note the separation of the right intrahepatic ducts on MRCP (arrowheads).

**Figure 6:**
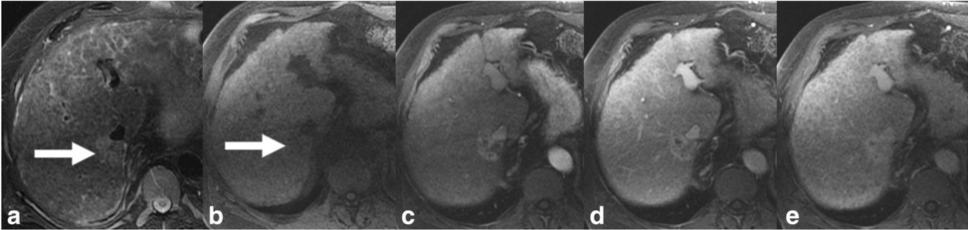
**Cirrhosis of liver with iCCA.** T2-weighted axial **(a)**, T1-weighted axial **(b)** and post gadolinium enhanced T1-weighted axial arterial phase **(c)**, portal venous phase **(d)** and delayed phase **(e)** images showing iCCA as an iso- to hyperintense lesion (arrow) in posterior right lobe with typical arterial phase rim like enhancement and progressive central enhancement through delayed phase without any washout. The liver parenchyma is heterogeneous and nodular consistent with cirrhosis.

**Figure 7:**
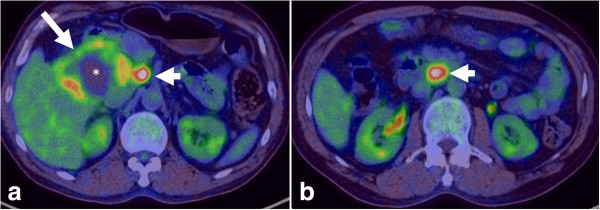
**PET-CT of iCCA.** Axial PET-CT images showing a large FDG-avid iCCA (arrow) with central photopenia (*) indicating necrosis/fibrosis with FDG-avid portal lymph node (**a**, arrow head) and aortocaval lymph node (**b**, arrow head) consistent with lymph node metastases.

The iCCA also known as peripheral cholangiocarcinoma (Figures 2, 3, 4, 5, 6, and 7) occur distal to second order bile ducts within the hepatic parenchyma and is the second most common intrahepatic primary tumor. iCCA arise from biliary epithelium at any portion of the intrahepatic biliary system, from the mucin-producing cylindrical cells lining the segmental bile ducts or cuboidal cholangiocytes without mucin production that line the bile ductules [25]. The histopathology can resemble adenocarcinoma of almost any organ. iCCA are highly infiltrative and contain areas of fibrosis, necrosis and mucin. Active tumor growth is frequently found at the periphery of the tumor. The most common subtype is the mass-forming (Figures 2, 3, 6 and 7), accounting for 80% of the iCCA and this subtype spreads via venous and lymphatic vessels [26]. The periductal-infiltrating type spreads mainly longitudinally along and within the bile duct often resulting in dilatation of the peripheral ducts (Figure 5). This also tends to spread along the lymphatics. The intraductal-growth type (Figure 4) proliferates towards the lumen and often has papillary growth characteristics. Papillary intrahepatic CCA occasionally produces abundant mucin that can result in massive expansion of the duct and present as a cystic mass mimicking cystadenocarcinoma [27]. The infiltrating type with mass forming features has the worst prognosis among the intrahepatic types [22]. The rarest form of intrahepatic CCA is the superficial spreading type and has a better outcome [28]. iCCA presents with non-specific symptoms such as abdominal pain, weight loss and night sweats and uncommonly jaundice.

### Imaging

The imaging features of iCCA are dependent on their size and proportion of fibrosis, necrosis and mucin content.

#### Ultrasound

Intrahepatic cholangiocarcinoma has variable echogenicity on sonography but tends to be hyperechoic [29] and simulate other masses (Figure 2). The internal architecture of the tumor is usually homogeneous, but it can be heterogeneous, depending on the amount of fibrous tissue, mucin and calcification. With contrast enhanced ultrasound, iCCA may show washout and mimic HCC [30] and therefore is not recommended as the sole imaging technique for conclusive diagnosis of HCC [31].

#### CT

On CT, intrahepatic cholangiocarcinomas can be well defined or infiltrative, and they lack the fibrous capsules found in HCC [29-32]. iCCA are typically hypo- or iso-attenuating relative to normal hepatic parenchyma on unenhanced CT with most remaining hypoattenuating during the arterial and portal venous phases with enhancement only in the delayed phase (Figures 3, 4, 5 and 6). These findings reflect their hypovascular desmoplastic composition [13,33]. The periphery of the malignant mass, in which active tumor growth is found, enhances rapidly after contrast enhancement and becomes isodense or hypodense during the portal phase [34]. Fibrous tissue, in the central area of the tumor, does not enhance during the early phase but becomes hyperdense during the delayed phase to 20 minutes later [34,35]. The central portion of the tumor remains hypodense, however, with necrotic or mucin-producing tumors. The degree of enhancement varies among tumors and some small mass-forming intrahepatic CCA are arterially enhancing, mimicking HCC. The use of delayed phase increases diagnostic confidence in nearly half of the cases [32].

Tumor enhancement on delayed CT imaging has actually been correlated with outcome. Asayama and colleagues [36] found that tumors that exhibited delayed enhancement on CT in more than two thirds of their volume had an increased amount of fibrous stroma and perineural invasion and were associated with a worse prognosis. The dense fibrotic nature of the tumor may result in capsular retraction in about one-fifth of cases [37-39].

#### MRI

The MR imaging features of iCCA differ according to its pattern of growth. Mass-forming ICC is irregularly marginated and demonstrates signal intensity depending on the degree of fibrosis, necrosis, hemorrhage and mucin within the tumor [13] (Figures 3, 5 and 6). iCCA is typically hypo to isointense on T1-weighted (T1W) and variably hyperintense on T2-weighted (T2W) imaging. The amount of T2W hyperintensity is also determined by the pathological subtype: the scirrhous subtype demonstrates relatively lower signal intensity as compared to a well-differentiated adenocarcinoma, owing to the fact that it is more fibrous with less mucin and necrosis [9]. Occasionally, CCA can be isointense to hepatic parenchyma on both T1W and T2W imaging [40].

Following the intravenous administration of gadolinium chelates, CCA typically show minimal or heterogeneous enhancement at the tumor periphery on early images, with progressive central enhancement on subsequent delayed images [41] (Figures 3, 5 and 6) owing to the fibrous composition [15]. The area of tumor with early enhancement indicates active growth. Progressive and prolonged delayed enhancement is seen in areas of fibrosis where there is decreased arterial blood supply with loose connective tissue and abundance of intercellular matrix [9].

Transient hepatic intensity differences and capsular retraction may also be observed [42]. In addition, the enhancement pattern is slow and peripheral in the arterial phase, with progressive concentric enhancement over time like a ring [43]. Encasement of hepatic vessels without thrombosis and hepatolithiasis are not uncommon findings.

When hepatobiliary-specific contrast agents are used, surrounding hepatic parenchyma enhances more because of the hepatocyte uptake. As a result, iCCA will appear relatively more hypointense (Figure 3) and early experience had indicated that this feature allows for better lesion demarcation [44]. Both MRI and CT are comparable for detection of satellite lesions.

Differentiating iCCA from HCC can be difficult especially if there is absence of progressive enhancement pattern [45]. This is an important clinical issue since iCCA can also occur in cirrhotic livers. Laboratory tests are also useful in establishing the diagnosis, because alpha-feto protein (AFP) levels are usually normal or only slightly elevated in intrahepatic cholangiocarcinoma compared with HCC. CA 19–9 may be increased. Up to 81% of iCCA are characterized by a progressive contrast uptake throughout the arterial and venous phase and later in the delayed phase without a prompt washout on both CT and MRI [45]. Hemangiomas also show progressive contrast enhancement but it has characteristic peripheral nodular enhancement quite different from iCCA. In one of the largest study, none of the iCCA showed the characteristic imaging features of HCC [45]. A subtype known as cholangiocellular carcinoma with mixed histological features, including HCC and CCA may show HCC imaging characteristics leading to diagnostic problems. A percutaneous biopsy is therefore required for final diagnosis in cases of lesions that show atypical features to confirm diagnosis especially if they are candidates for surgical resection. Histological differentiation from metastases can be improved by immunoprofiling with a combination of cytokeratin (K) 7 and K20 immunohistochemical staining [46].

#### PET

PET provides metabolic information on tumors, and with regards to CCA, the high glucose uptake of bile duct epithelium enables detection of tumors as small as 1 cm but is less helpful for infiltrative periductal tumours [47,48]. The specificity of PET for the detection of mass-forming intrahepatic CCA >1 cm in diameter has been reported as 85-95% with a sensitivity of 100% [49]. A drawback is its inability to differentiate malignant from benign lesions limiting its use as a standalone imaging modality in diagnosing CCA [47]. However, it has been documented that PET can complement cross-section imaging in identifying occult distant metastases (Figure 7) and detection of recurrence with previously treated/resected CCA. Early data had also indicated that information from PET scans could lead to a change in surgical management due to detection of unsuspected metastases [48].

### Staging and treatment

The prognostic factors include tumor number and differentiation, lymph node metastases and vascular invasion. Regional lymph node metastases are an independent predictor of survival. Three staging systems are available for cholangiocarcinoma: The American Joint Cancer Committee/Union for International Cancer Control (AJCC/UICC) TNM staging system [50], the liver cancer study group of Japan (LSCGJ) staging system [22] and the National Cancer center of Japan (NCCJ) staging system [51]. However, these staging systems, although not significantly different cannot provide prognostic information and are also not able to stratify the patients to treatment arms [8].

Surgical resection of iCCA is associated with high rates of tumor recurrence and short survival periods. Positive margins, lymph node metastases and cirrhosis are associated with reduced survival time [21]. Liver transplant is not considered a good option as the 5-year rate of tumor recurrence is about 70% with a median time of disease free survival of only 8 months [52]. Palliative treatment options include radiofrequency ablation (RFA), trans arterial chemoembolization (TACE) and trans arterial radio embolization (TARE) [53,54]. Systemic chemotherapy with combination of gemcitabine and cisplatin is probably the treatment standard in patients with inoperable iCCA as it has been shown to prolong survival times [55].

### Perihilar CCA (pCCA)

**Figure 8:**
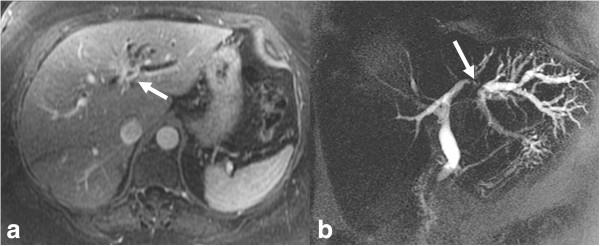
**Infiltrating pCCA.** Axial post contrast enhanced T1-weighted MR image **(a)** and MRCP **(b)** images demonstrating an enhancing stricture involving the left hepatic duct (arrow) with upstream dilatation of the left hepatic ducts.

**Figure 9:**
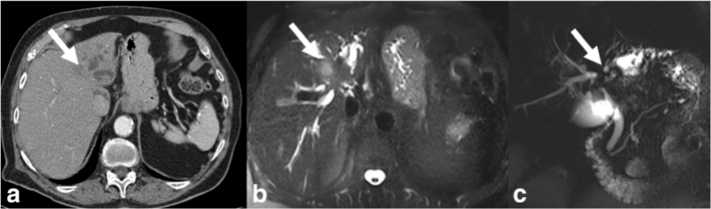
**Peripheral cholangiocarcinoma with involvement of confluence.** Infiltrating pCCA of the left hepatic duct (arrow) isodense to liver parenchyma on axial contrast enhanced CT **(a)** with dilation of the left hepatic ducts. The ductal thickening is hyperintense (arrow) on T2-weighted MRI image **(b)** with extension to the confluence causing mild dilatation of the right hepatic ducts demonstrated better on MRCP **(c)**.

**Figure 10:**
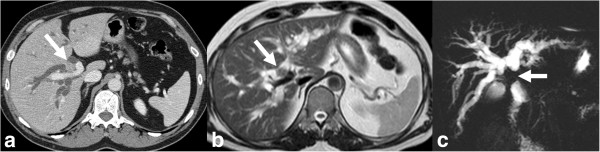
**CCA of common hepatic duct with involvement of confluence.** Contrast enhanced CT **(a)**, T2-weighted MRI **(b)** and MRCP **(c)** images demonstrating thickened and enhancing common hepatic duct (arrow) with involvement of the confluence and upstream dilatation of the intrahepatic ducts. The ductal thickening appears hypointense (arrow) to the surrounding dilated bile ducts on T2-weighted MRI image **(b)**. The involvement of the confluence is demonstrated better on MRCP.

**Figure 11:**
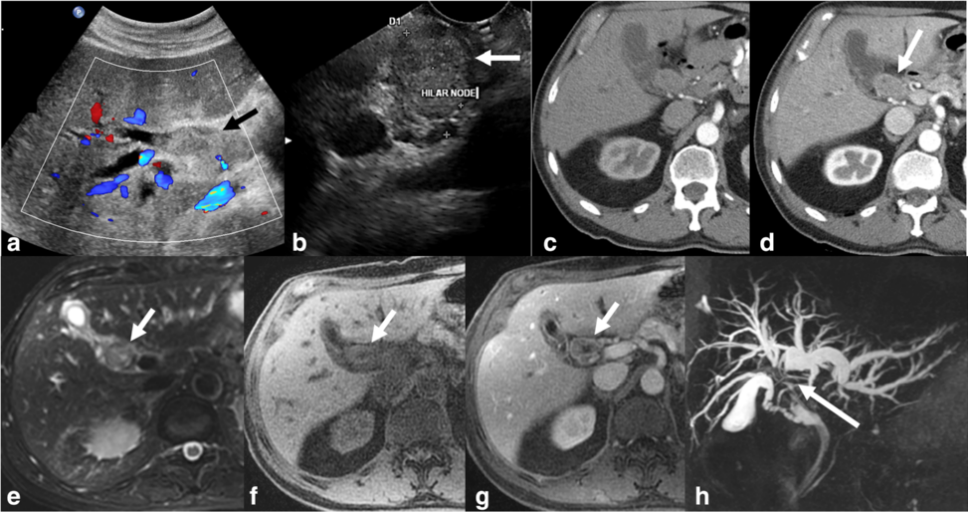
**CCA of common hepatic duct with involvement of confluence.** Ultrasound with color flow overlay **(a)** showing an echogenic mass (arrow) filling the common hepatic duct with upstream dilatation of the intrahepatic ducts. EUS image **(b)** showing a hilar lymph node and was sampled positive for carcinoma. Contrast enhanced CT in arterial phase **(c)** and portal venous phase **(d)** showing enhancing mass (arrow) within the duct and no major vascular involvement. The ductal mass is hyperintense on T2-weighted image **(e)** and hypointense on T1-weighted image **(f)** and shows concentric post contrast enhancement **(g)**. MRCP **(h)** shows the confluence invasion and non- visualization of the common hepatic duct.

**Figure 12:**
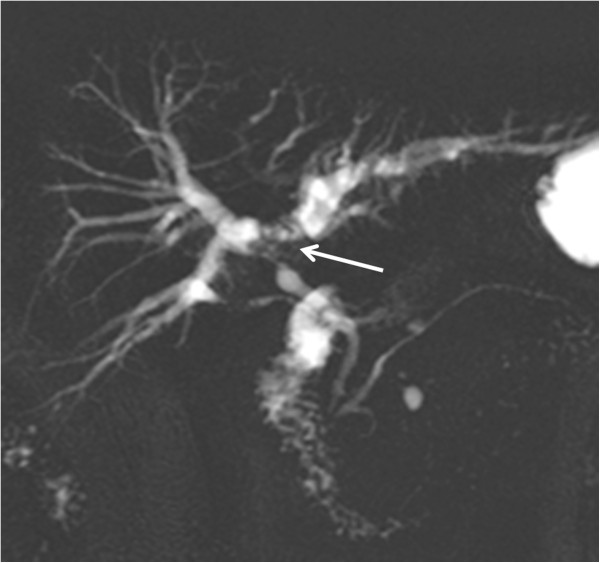
**MRCP of a classical Klatskin’s tumor.** The confluence, proximal hepatic ducts and proximal common hepatic duct are strictured (arrow). The common bile duct is of normal caliber.

**Figure 13:**
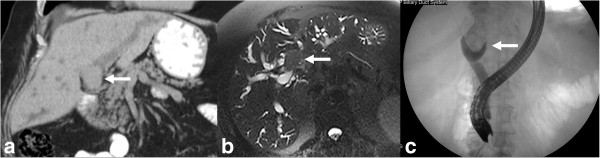
**Hilar CCA presenting as an intraluminal mass with biliary obstruction.** The mass is isodense (arrow) on the coronal non-contrast enhanced CT **(a)** and hypointense on axial T2-weighted image **(b)** with upstream dilatation of the intrahepatic ducts. ERCP **(c)** shows a filling defect (arrow) representing the mass extending into the common bile duct with no filling of the intrahepatic ducts.

The pCCA develops anywhere from the second order biliary ducts to the common bile duct above and at the site of cystic duct origin (Figures 8, 9, 10, 11, 12 and 13). Klatskin’s tumors (Figure 12) are those pCCA that occur at the confluence of right and left hepatic ducts and the proximal common hepatic duct. Macroscopically they can be nodular, sclerosing (periductal infiltrating) and papillary subtypes [56]. Sclerosing or periductal infiltrating CCA is the most common type and papillary adenocarcinomas are rare but have the best prognosis among the CCA [57]. Most nodular and sclerosing tumors are well to poorly differentiated tubular adenocarcinomas that contain mucin glands lined by cuboidal epithelium and abundant fibrous stroma.

Sclerosing CCA do not usually form a mass but grow along the bile duct to produce a concentric thickening of the bile duct that manifests as a poorly defined stricture that eventually produces complete obliteration of the duct lumen [42]. Nodular CCA arises in the mucosa and grows within the lumen and then penetrates the bile duct wall. The nodular growth pattern with desmoplastic reaction results in a hard mass with well-defined margins that grows and almost always causes biliary obstruction [1]. Papillary tumors contain numerous papillary infoldings supported by fibrovascular stalks and grow intraluminally. They do not tend to invade bile duct wall and therefore have a better prognosis [58]. pCCA typically spreads by perineural invasion and lymphatic metastasis [8]. Lymphatic metastasis occurs most commonly to porta-caval, superior pancreaticoduodenal, and posterior pancreaticoduodenal lymph nodes. The liver parenchyma, the gastrohepatic and hepatoduodenal ligaments are commonly invaded by pCCA.

Most common clinical presentation is jaundice and it occurs in 90% of patients and about 10% may present with cholangitis. Systemic symptoms including malaise, abdominal discomfort, nausea, anorexia and weight loss may be seen in about half of patients [59,60]. Depending on the location, they may cause chronic biliary obstruction leading to atrophy of segments and lobes. Lobar hepatic atrophy in association with biliary dilatation strongly suggests pCCA. Unilateral hepatic lobe hypertrophy with contralateral hepatic lobar atrophy known as hypertrophy-atrophy complex occurs when there is unilobar biliary obstruction often with vascular encasement [61].

### Diagnosis

The diagnosis of pCCA is challenging and a multimodality approach has to be taken in most of the cases. Imaging is critical in diagnosis and management of pCCA. The role of modern imaging, after excluding metastatic disease, is to determine the T stage that will guide surgical management. Accurate reporting with assessment of the presence of lobar atrophy, sectoral or main portal vein involvement and tumor extension into secondary biliary radicals will help guide the hepatobiliary surgeon to determine preoperatively the extent of local tumor involvement and the surgical resection to be performed. In addition, determination of the presence of distant disease, lymphadenopathy, or satellite hepatic metastases is paramount.

Tumor markers can be useful in combination with other imaging tests and in cases of indeterminate biliary strictures. In patients with PSC, CA19-9 has sensitivity and specificity of 79%and 98% respectively at serum concentration >129U/ml [62]. In patients without PSC, a CA19-9 > 100U/ml has sensitivity of 76% and a negative predictive value of 92% compared to those with benign strictures [24]. It should be noted however that nearly 10% of the population may not secrete CA19-9 and occasionally the tumor may not express tumor marker [63]. Differential diagnosis of pCCA includes lymph nodes, benign strictures and rarely lymphoma or sarcoma involving the bile ducts [37-39].

#### Ultrasound

Ultrasound is often the initial imaging study in patients presenting with obstructive jaundice. This modality is useful in ruling out benign causes of bile duct obstruction, including choledocholithiasis, and is reliable in demonstrating the intrahepatic ductal anatomy and the proximal level of obstruction (Figure 11). The sensitivity and accuracy of ultrasound for diagnosis of extrahepatic CCA is 89% and 80-95% respectively [33,64]. Often the strictures and the mass are not visible on ultrasound; however lesions that form masses and invade surrounding liver parenchyma or involve portal vessels can be demonstrated on ultrasound. Ultrasound findings are useful in determining the next best imaging modality for complete assessment of the tumor.

#### CT

CT has become the non-invasive diagnostic test of choice for evaluation and staging of pCCA allowing for extent of local invasion, depiction of level of biliary obstruction (Figure 11) and review of the abdomen and pelvis for distant spread [13].

Multidetector CT has 78.6%-92.3% accuracy for diagnosis of extrahepatic CCA [34] but has strong tendency to underestimate the longitudinal extension of the tumor [65]. The accuracy for detection of portal vein and arterial involvement has been reported to be as high as 87% and 93% respectively [66]. The accuracy of CT in the assessment of resectability has been reported as 60-88% with negative predictive values of 85-100% [67]. However, its sensitivity in the detection of regional lymphadenopathy is only 54% and CT tends to underestimate the extent of proximal tumour [68]. Also, streak artifacts’ and secondary inflammatory changes that can occur when a stent is placed limits evaluation with CT [69].

CT hepatic arteriography, CT portography and CT venographic images provide a detailed pre-operative vascular roadmap comparable to that provided by catheter angiography facilitating accurate surgical planning [13]. CT cholangiography provide details of the biliary anatomy and are considered in cases where MR imaging is contraindicated or unavailable. CT cholangiography is superior to conventional CT or US and equal to ERCP for diagnosis of pCCA [66]. A potential limitation of CT cholangiography is the dependence on the secretory function of the biliary system that may be compromised in patients with high-grade obstruction or significantly elevated bilirubin levels [13].

#### MRI and MRCP

Diagnosis of the periductal infiltrating CCA can be difficult owing to the infiltrative nature of the tumor and absence of a mass-like lesion. Typically, the main imaging features are biliary duct obstruction as evident by proximal ductal dilatation, periductal thickening and enhancement (Figure 12) [15,41]. As mentioned previously, the intra-ductal mass type is rare, it typically manifests as an enhancing intraductal mass associated with proximal ductal dilation [15].

**Figure 14:**
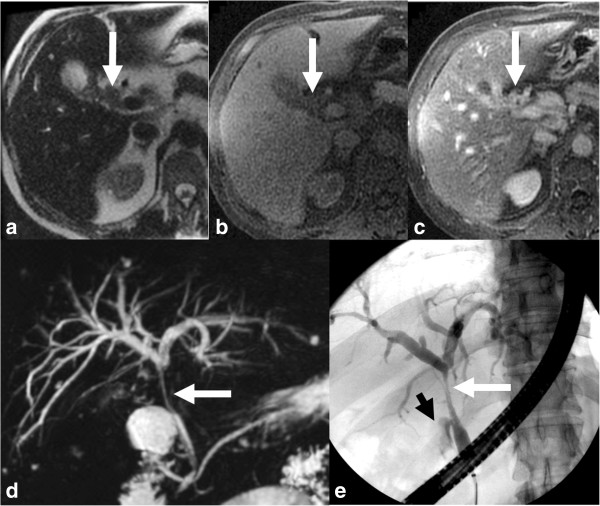
**Perihilar CCA arising from the cystic duct and proximal gall bladder (arrows) with invasion of common hepatic duct.** The pCCA is mildly hyperintense on T2-weighted image **(a)**, hypointense on T1-weighted image **(b)** and shows post contrast enhancement **(c)**. Both MRCP **(d)** and ERCP **(e)** demonstrate the stricture of the common hepatic duct till the confluence above and cystic duct insertion below. The gall bladder is not visualized on ERCP and the irregularity of the proximal cystic duct (arrowhead) is due to the tumor.

MRCP is an accurate method for anatomically mapping the biliary tree. It does not require biliary instrumentation. MRCP is now considered the radiological modality of choice for evaluating patients with suspected CCA [43]. MRCP should be ideally performed before decompressing the biliary tree. With MRCP, there is better assessment of the extent of peripheral ductal involvement as compared to ERCP (Figures 13 and 14). This is because ducts proximal to an obstructing tumor may not adequately fill during ERCP [43] (Figure 13). The reported accuracy in determining the extent of bile duct tumors ranges from 71% to 96% [43]. In addition, acquisition of 3D data sets provides information useful for preoperative management and surgical planning. MRI with MRCP is the imaging technique of choice in many centers secondary to its excellent soft tissue contrast that is particularly useful for evaluation of infiltrating ductal tumors [69]. MRI with MRCP has an accuracy of 66% for detection of lymph node metastases [70], 78% sensitivity and 91% specificity for portal vein invasion [71] and 58-73% sensitivity and 93% specificity for hepatic arterial invasion [72].

More recently, hepatobiliary-specific contrast agents have been developed to overcome the limitations of non-specific extracellular fluid agents [73]. Since their initial approval in Europe and Asia as early as 2005, they have been widely available for the detection and characterization of focal hepatic lesions [44]. Hepatobiliary-specific contrast agents are divided into two main categories: manganese-based (mangafodipir trisodium, Teslascan®) and gadolinium-based (gadobenate dimeglumine, MultiHance® and gadoxetic acid, Primovist® in Europe and Eovist® in the United States) agents. Teslascan is given as a slow infusion hence preventing acquisition of dynamic images during the arterial and portal venous phases [73]. This product has been removed from the United States market from September 2004 but remains in use in Europe and Asia [74].

Gadolinium-based hepatobiliary-specific agents initially distribute in the extracellular fluid compartment, sharing the imaging properties of extracellular fluid agents during the arterial and portal venous phases. However, they are actively taken up by hepatocytes and excreted into the bile. Hence, gadolinium-based hepatobiliary-specific agents provide the dual benefit of dynamic imaging capability as well as delayed hepatobiliary phase imaging [73].

In addition, fluid collections, ascites and fluid-containing structures that can obscure findings on conventional T2W MRCP are characteristically less conspicuous with T1W sequences used in contrast- enhanced MR cholangiography, which may help in delineation of the biliary tree [73].

#### ERCP and percutaneous transhepatic cholangiography (PTC)

Both ERCP and PTC are invasive techniques that assess biliary ducts and have the added advantage of obtaining samples for histology. Due to their invasive nature especially with ERCP, which is more commonly performed, there is risk of complications such as post-ERCP pancreatitis, cholangitis and bleeding. Vascular injury and death can occur with both techniques. The sensitivity and specificity of cholangiography is about 75% with an accuracy of 95% for diagnosis of pCCA [75]. The possibility of obtaining biopsy and brush cytology is promising but is not a successful tool as desmoplastic reaction limits the number of cells obtained by cytology. The routine brush cytology has 9%-24% sensitivity and 61%-100% specificity for CCA [76]. A repeat brushing may improve the sensitivity to 44% [77]. Advanced cytologic techniques including digitized image analysis (DIA) and fluorescence in situ hybridization (FISH) are used to increase the sensitivity of cytology [78]. DIA increased the sensitivity to 39% in one study [79] and FISH increased the sensitivity to 47% [80], suggesting the moderate gain obtained with these techniques. However, DIA and FISH improves sensitivity in patients with PSC and elevated CA 19–9 (>20U/mL) to 56% and 86% respectively [81]. Per oral cholangioscopy and intraductal ultrasound are emerging techniques and larger experience with these new techniques is awaited.

#### Endoscopic ultrasound (EUS)

Recently, EUS has emerged as an important modality in the diagnosis of CCA [82]. EUS-guided FNA (EUSFNA) can be used for assessing the nature of biliary strictures and for providing information on the extent of periductal disease and the presence of lymph node metastases (Figure 11). It is gaining rapid popularity due to its greater sensitivity for detecting malignancy in distal tumors than does ERCP with brushings. EUSFNA has a specificity of 100% and a sensitivity of 43-86% depending upon location of the CCA [83]. In addition, EUSFNA also avoids contamination of the biliary tree, which can occur with ERCP [84]. Early data had shown that information from EUSFNA had changed the management in patients with previously non-diagnostic ERCP [82]. However, its use in imaging and staging proximal bile duct lesions is uncertain with clinical experience still limited [85]. At the same time, EUSFNA of primary lesions in potential candidates for treatment with curative intent is still discouraged due to the risk of peritoneal seeding [84].

#### PET

Experience with PET in extrahepatic CCA is limited. In patients with areas of inflammation along the bile duct associated with PSC, interpretation can be difficult as areas of inflammation may have increased uptake and desmoplastic areas of low cellularity may lead to possible false negatives.

### Staging and treatment

Accurate evaluation of tumour extent is necessary for optimum management. The classic Bismuth-Corlette classification [86] for assessment of biliary tree involvement has been incorporated into a new surgical staging system that considers tumor size (>1 cm, 1-3 cm or > =3 cm), tumor morphology, degree of specific location of hepatic artery and portal vein encasement (vessel involvement > 180 degrees indicates encasement), volume of the potential liver remnant, presence of other liver diseases, status of lymph nodes and distant metastases.

Surgery remains the mainstay of curative therapy, the aim being complete tumor excision with negative histological margins, relief of obstruction and re-establishment of bilio-enteric communication [33]. The following complications preclude curative resection: involvement of the right or left main hepatic duct to the level of the secondary biliary radicals; atrophy of one hepatic lobe with contralateral portal vein branch encasement or contralateral secondary biliary radical involvement; vascular encasement or invasion (proper hepatic artery, bilateral hepatic arteries, main portal vein); and metastases to lymph nodes, peritoneal cavity or distant organs [33]. Radical resection of pCCA has 5%-10% perioperative mortality rate [87]. Portal vein embolization is a valuable pre-operative measure when extensive liver resections are performed. The average 5-year survival rates following resection are 25%-40% [87] and the favourable outcome are associated with R0 resection, no lymph node metastasis, absence of perineural invasion, and well differentiated histological grade [26].

Liver transplantation is not routinely performed for pCCA but can increase survival in selected patients wherein resection is not an option secondary to locally advanced disease. Criteria for liver transplantation for patients without PSC are: tumor less than 3 cm radial diameter, no intrahepatic or extrahepatic metastases and unresectability. In patients with PSC, the criteria are a tumor less than 3 cm with no evidence of metastases [6]. Radiation and chemotherapy have shown no benefit [88]. Palliative surgery is often performed to relieve symptoms of obstruction [9] and jaundice relief with biliary drainage.

### Distal CCA (dCCA)

**Figure 15:**
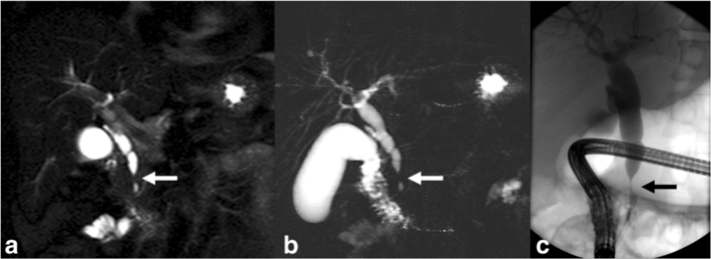
**Distal CCA.** Common bile duct stricture due to grade 4 invasive carcinoma. Coronal T2-weighted image **(a)** and MRCP **(b)** image showing a short segmental narrowing (arrow) with proximal dilatation. ERCP **(c)** showing a short segmental stricture representing the invasive CCA.

This subtype develops anywhere in the common bile duct between the cystic duct origin and the ampulla of Vater without its involvement (Figure 15). These are separate from ampullary carcinomas. dCCA are thought to arise from intraductal papillary neoplasm or biliary intraepithelial neoplasia [28]. Histologically they are predominantly well to moderately differentiated adenocarcinomas. When these arise in distal bile duct within the pancreas, it’s difficult to distinguish it from cancer of the head of the pancreas. Clinically, patients present with symptoms of painless jaundice and cholangitis. Lymph node metastases are less common than in the pCCA type.

### Diagnosis and staging

Distinct features and imaging characteristics of this type are not well known as these are frequently clubbed with pCCA and referred to as extrahepatic CCA.

**Figure 16:**
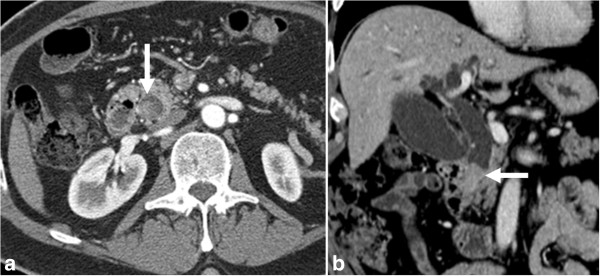
**Distal CCA presenting as a polypoid mass with obstructive jaundice.** Contrast enhanced CT axial **(a)** and coronal reconstruction **(b)** showing a soft tissue density filling defect in distal common bile duct (arrow) representing the invasive grade 3 adenocarcinoma.

Ultrasound is useful to demonstrate the obstruction level at the lower end of the bile duct and proximal dilatation. CT and MRI with MRCP may demonstrate thickening and/or stricturing of bile duct (Figure 15) with proximal duct dilatation and sometimes a mass (Figure 16). The imaging can also help delineate invasion of vessels and the pancreas. ERCP is specific and has high positive predictive for dCCA [89]. EUS is important in the pre-operative evaluation of dCCA and EUS-FNA is very specific for predicting unresectability [90]. Intraductal ultrasonography may be useful in evaluation of invasion of surrounding structures. Tumor depth invasion, lymph node metastases, perineural, microscopic vascular invasion and pancreatic invasion are significant predictors of survival [91-94].

### Treatment

Surgery is usually a pancreaticoduodenectomy (Whipple’s procedure). The overall 5 –year survival rate of patients with dCCA after complete resection is 27% with a median survival of 25 months [21]. Nearly 2/3^rd^ of patients with dCCA who undergo surgery have involvement of lymph nodes [21]. Negative tumor margins are the most important predictor of patient survival. Palliative treatment comprises of relief of biliary obstruction with or without chemotherapy.

### Gallbladder carcinoma (GbCA)

Gallbladder carcinoma (GbCA) is an uncommon but highly lethal malignancy. GbCA is defined as cancer arising from the gallbladder and the cystic duct [1]. Anatomic factors promote early local invasion, with the ease by which this tumor invades the liver and the biliary tree contributing to its high mortality [95]. It also exhibits a propensity for invasion to lymph nodes, hematogenous spread and capacity to implant on peritoneal surfaces [96]. It is also often diagnosed late due to its non-specific nature of symptoms and signs common to other benign diseases such as cholelithiasis or chronic cholecystitis [96]. The prognosis is poor with a reported 5-year survival rate of less than 5% in most large series [95]. Majority of cases of GbCA are discovered incidentally at surgical exploration for benign gallbladder disease [97].

Fewer than 5000 are diagnosed each year in the United States with the incidence rate of 1 to 2 per 100,000 [98]. However, there is again a prominent geographic variability in the incidence that correlates well with the prevalence of cholelithiasis. For example, relatively high rates are seen in South American and North Asian countries, and these populations all share a high prevalence of gallstones and/or Salmonella typhi infection, both recognized risk factors [99]. The risk also seems higher in those with larger gallstones; Misra et al. [100] found that patients with stones larger than three cm had a ten-fold higher risk of GbCA compared to those with stones less than 1 cm.

Other risks factors include increasing age, female gender (with women affected two to six times more often than in men) [101], chronic cholecystitis, porcelain gallbladder, gallbladder polyps, primary sclerosing cholangitis and a congenital anomalous pancreaticobiliary duct junction [102]. Lifestyle factors such as obesity, diabetes and smoking are also contributory [20,95]. Exposure to chemicals used in the rubber, automobile, wood finishing, and metal fabricating industries have been associated with an increased risk of GbCA as well [95].

GbCA occurs from dysplasia and metaplasia of the epithelial lining of the gallbladder. Gastric metaplasia is the most common metaplasia in gallbladders [3] and intestinal metaplasia occurs with increasing age and in association with gallstone disease [4]. Squamous metaplasia tends to be associated with gallstones and can lead to squamous dysplasia or squamous cell carcinoma [4]. Adenomas occur in 0.3% -0.5% of the population and can be pedunculated, sessile, single or multiple and are often smaller than 2 cm [4]. The risk of malignant transformation increases with the size of the adenoma and the amount of papillary pattern. Approximately 98% of GbCA are of epithelial origin, with more than 90% identified as adenocarcinomas. Adenocarcinomas may be well, moderately, or poorly differentiated depending on the degree of gland formation [95]. The remaining subtypes include adenosquamous or squamous cell carcinoma, small cell neuroendocrine tumors, sarcoma, and lymphomas [97]. Most of these tumors originate in the gallbladder fundus (60%) with the remainder in the body (30%) and neck (10%) [91]. Rare non-epithelial tumors include sarcomas, lymphomas, carcinoid tumors, and metastases.

### Diagnosis

Clinical diagnosis of GbCA is challenging due to lack of specific signs and symptoms and therefore diagnosis is made quite late into the disease or as an incidental finding after cholecystectomy done for cholecystitis or other reasons. Most of the patients present with right upper quadrant abdominal pain. Weight loss, anorexia, nausea and vomiting are commonly associated [1]. Raised serum carcinoma embryonic antigen (CEA) levels may be useful to improve diagnosis.

**Figure 17:**
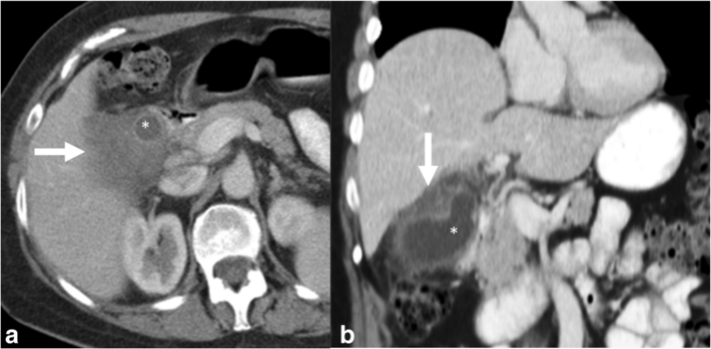
**Gall bladder carcinoma.** Contrast enhanced axial CT image **(a)** and coronal reformat **(b)** showing hypodense thickening of the gall bladder wall representing the carcinoma (arrow) with involvement of the adjacent liver. The thickening covers more than half of gall bladder lumen (*).

**Figure 18:**
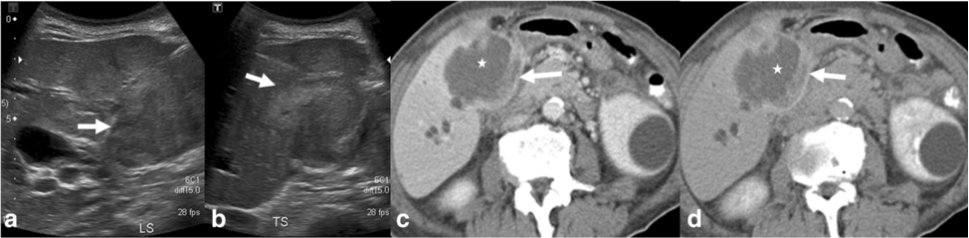
**Gall bladder carcinoma.** The gallbladder and gall bladder fossa is replaced by a large heterogeneous mass of mixed echogenicity on ultrasound **(a, b)** and heterogeneous hypodense mass (*) on contrast enhanced CT with enhancement of the fibrous stromal component (arrow) of gallbladder carcinoma during the portal venous **(c)** and delayed phases **(d)**.

**Figure 19:**
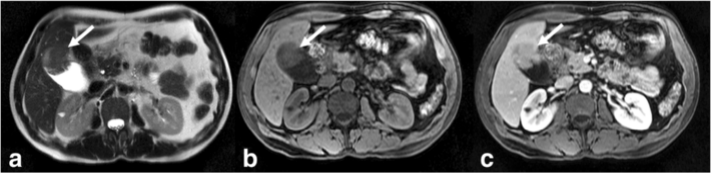
Gall bladder carcinoma arising from the fundus of the gall bladder (arrow) seen as an iso to hyperintense mass on T2-weighted (a) and hypointense mass on T1-weighted (b) images and shows enhancement on post gadolinium enhanced image (c).

Imaging studies may reveal a mass replacing the normal gallbladder, diffuse or focal thickening of the gallbladder wall (Figures 17 and 18), polypoid mass (Figure 19) within the gallbladder lumen or as a gallbladder fossa mass [103]. Mass replacing the gallbladder fossa is the most common presentation (Figure 17). Adjacent organ invasion, primarily involving the liver and biliary obstruction is often present at diagnosis. Periportal and peripancreatic lymph nodes, hematogenous and peritoneal metastases may also be seen [95]. About 25% of GbCA present as an intraluminal mass and they tend to have better prognosis as they are usually confined by the muscularis propria. GbCA presenting as focal or diffuse mural thickening is the least common and most difficult to diagnose. Benign conditions that are associated with diffuse wall thickening including acute and chronic cholecystitis, adenomyomatosis, hepatitis and inadequate bladder distension are more common.

#### Ultrasound and EUS

Ultrasonography is most often the first imaging modality in investigating gallbladder disease due to its relatively low cost and ease of availability [96]. However, in the case of GbCA, although ultrasonography can detect late-stage tumor with high sensitivity (Figure 9), its use is limited in early lesion diagnosis and staging [103]. This limitation can be overcome by combining endoscopy with ultrasound (EUS) and in recent years, endoscopic ultrasound has gained increasing popularity in assessment of GbCA. This technique enables assessment of the depth of tumor invasion into the wall of the gallbladder and presence of lymphadenopathy at the porta hepatis and peripancreatic regions. It can also provide a means of obtaining bile for cytological analysis that has been found to have 73% sensitivity for the diagnosis of GbCA [104].

Discontinuous thickening of the gallbladder mucosa, diffuse thickening of the gallbladder wall (>12 mm), mural calcification, a mass protruding into the lumen, a fixed mass in the gallbladder, and loss of the interface between the liver and the gallbladder are all signs commonly associated with gallbladder cancer [105]. Nodular and papillary GbCA is more likely to be associated with a mass and easily detected as compared to infiltrative carcinoma. Conventional US appears to be quite reliable in the detection of masses and the extent of hepatic invasion, but it is limited in its ability to detect lymph node and peritoneal disease. Polypoid carcinomas have homogeneous tissue texture and are fixed to the gallbladder at their base. Small polypoid carcinomas are difficult to differentiate from a cholesterol polyp, adenoma, adherent stone, sludge or a blood clot.

#### CT

CT is a better modality for evaluation of thickness of the portions of the gallbladder wall that are obscured on ultrasound. Wall thickening remains diagnostically challenging as it mimics the appearance of more common inflammatory conditions of the gallbladder. Marked wall thickening (>1.0 cm) with associated mural irregularity or significant asymmetry (Figure 17) should raise concerns for malignancy [95]. Diffuse symmetric wall thickening is more likely to suggest a non-neoplastic process [106].

GbCA are usually hypodense on unenhanced CT with up to 40% showing hypervascular foci of enhancement equal or greater than that of the adjacent hepatic parenchyma [106]. Contrast enhancement may be retained in fibrous stromal components of gallbladder carcinoma during the portal venous and delayed phases – this can potentially aid in differentiating gallbladder carcinomas from hepatocellular carcinomas (which have a greater tendency to washout in these phases) (Figure 17).

Prevalence of lymphatic spread is high, progressing from the gallbladder fossa through the hepatoduodenal ligament to nodal stations near the pancreatic head. Masses around the common bile duct and pancreatic head may mimic a pancreatic head carcinoma [95].

The sensitivity of contrast enhanced CT (ceCT) in detecting gallbladder neoplasms has been reported to be as high as 90% and is particularly effective in detecting T2 or greater tumors [107]. Appearances on ceCT can include a low-attenuation mass, enhancing mass with ill-defined borders, eccentric gallbladder wall thickening or a fungating mass. The information obtained through ceCT is also critical in assessing resectability of gallbladder tumors; it provides valuable information on local and vascular invasion as well as hematogenous and lymph node metastases, although its reliability in staging lymph node disease is not always accurate.

#### MRI

On MRI, GbCA are usually hypo to iso-intense on T1W and moderately hyperintense on T2W sequences (Figure 19) with enhancement characteristics similar to that of CT [103]. MRI may be more useful relative to CT in the assessment of focal or diffuse mural thickening as it may be able to distinguish GbCA from benign entities such as adenomyomatosis and xanthogranulomatous cholecystitis. Rokitansky-Aschoff sinuses in adenomyomatosis are best visualized on T2W sequences [95] and lipid-laden macrophages found in xanthogranulomatous cholecystitis can be demonstrated as a drop-out in signal in opposed phase images.

MR angiography and MRCP can be added to facilitate the diagnosis of vascular and biliary infiltration that is essential before attempting curative resection. Any focal or eccentric stenosis, irregularity of the lumen or abrupt amputation is suggestive of invasion.

#### PET

An intense accumulation of 18 F-FDG in the region of the gallbladder suggests malignancy although it lacks specificity in differentiating primary gallbladder carcinoma from other malignant lesions such as HCC, CCA and metastatic disease [49]. In addition, benign inflammatory lesions can also accumulate FDG and result in false positive interpretations. PET however has a promising role in the detection of unsuspected metastases that may modify staging and therapy [96].

### Staging and treatment

GbCA are staged according to UICC/AJCC staging system [50]. Invasion of portal vein, hepatic artery or two or more extrahepatic organs is considered T4 disease. Surgery again, is the only potential curative therapy [108]. As mentioned above, GbCA is most often discovered incidentally at surgical exploration for benign gallbladder disease. The surgeon then has to exercise clinical judgment during the operation; completing the cholecystectomy alone, obtain an intra-operative frozen section of the gallbladder (which if positive would lead to a more extensive resection) or proceed with resection of the gallbladder along with a rim of liver tissue. Although an intraoperative frozen section can reliably indicate the presence of malignancy, it cannot reliably predict the depth of tumor invasion. External beam radiation therapy and systemic chemotherapy have improved survival in patients with negative resection margins [109].

If the cancer is locally unresectable, chemotherapy or chemoradiotherapy can be considered. There is no indication for radical surgery for the purpose of debulking and attempted resection should only be accepted if it is possible to achieve complete resection [110]. The goal of palliation in advanced gallbladder cancer is relief of pain and jaundice along with prolongation of life. Placement of endoscopic or percutaneous biliary prostheses may be performed [96].

### Ampullary carcinoma

The ampulla of Vater comprises the junction of the biliary and pancreatic ducts and is surrounded by the sphincter of Oddi; it traverses a dehiscence of the duodenal wall and terminates as the major duodenal papilla [111]. Ampullary carcinomas are defined as those that arise within this ampullary complex, distal to the bifurcation of the distal common bile and pancreatic ducts. The duodenal papilla is lined by intestinal mucosa, whereas the ampullary portions are covered by simple mucinous epithelium, as in the normal bile duct – malignancies of the ampulla can arise from these two cell types. Pancreaticobiliary type of differentiation is more common than intestinal [112]. Intestinal type is associated with better survival [7]. The ampulla is surrounded by the parenchyma of the pancreatic head and the duodenum and this area is called the periampullary region within 2 cm of the ampulla.

Ampullary carcinoma is rare, with an incidence rate of 4–6 per million [113]. However, it tends to show a better prognosis as compared to the aforementioned biliary malignancies because it can be detected at a relatively early stage owing to biliary obstruction resulting in jaundice. The intestinal type ampullary carcinoma is relatively more common, and its incidence can increase 200 to 300 fold among genetically susceptible groups such as patients with hereditary polyposis syndromes [114]. The average age at diagnosis of sporadic ampullary carcinomas is 60–70 years old with patients with an inherited polyposis syndrome presenting at an earlier age, due in part to surveillance programs [115].

Biliary dilatation is seen in 75% of cases and pancreatic ductal dilatation in 67% [116]. Histology of primary ampullary neoplasms tends to resemble adenomas and adenocarcinomas of intestinal origin rather than a pancreaticobiliary origin [117]. True ampullary cancers have a better prognosis compared to periampullary malignancies of pancreatic or extrahepatic biliary origin with higher resectability rates and a 30-50% survival rate [118].

### Diagnosis and imaging

Differentiating a primary ampullary carcinoma from the more prevalent periampullary malignancies can be challenging. It may not be possible to determine the tissue origin until resection and histopathological evaluation.

#### CT

**Figure 20:**
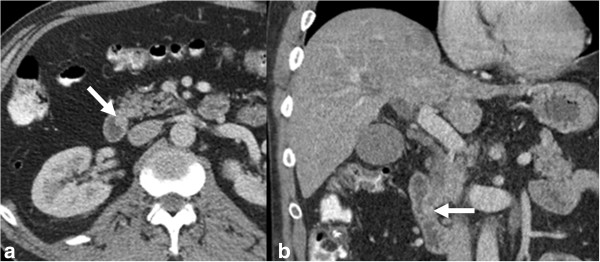
**Ampullary carcinoma.** Contrast enhanced axial CT **(a)** and coronal reformat **(b)** showing a small polypoid mass (arrow) representing carcinoma in the ampulla of the bile duct.

Although CT (Figure 20) can detect masses obstructing the distal common bile duct, it usually is not sensitive enough to allow visualization of small ampullary tumors within the duodenal lumen. CT also lacks the spatial resolution to determine exact extent of local invasion but is generally useful for assessing presence of lymphadenopathy and distant metastatic disease. Marked and abrupt dilatation of the distal bile duct or pancreatic duct in the absence of stones or pancreatitis is highly suggestive of ampullary carcinoma [111].

#### ERCP

In a patient with jaundice secondary to malignant bile duct obstruction, an ERCP is preferred as it permits biopsy and placement of a stent for biliary decompression if necessary. Although it allows visualization of the neoplasm, it cannot determine extent of local invasion. If an exophytic ampullary tumor is discovered, malignancy is highly suggestive if the mass is ulcerated or greater than 3 cm in size [119].

#### MRI and MRCP

Most appear as a discrete nodular mass at the distal margin of the pancreaticobiliary junction and are hypointense on T2W imaging [111]. The remainder can appear as irregular periductal thickening around the pancreaticobiliary junction or papillary bulging into the duodenum [111]. MRCP is a non-invasive method of imaging the pancreaticobiliary tree and is used in those who either cannot tolerate the more invasive ERCP or in whom a large tumor occludes the orifice of the duct hence preventing cannulation and duct opacification. The neoplasm appears as a filling defect within the duodenal lumen with characteristic delayed enhancement.

#### EUS

EUS is found to be as sensitive as ERCP and superior to CT for the detection of small ampullary tumors [120]. EUS accurately displays the depth of tumor invasion into the duodenum and local extension to adjacent structures. EUS has been found to be the most accurate modality to assess local staging of ampullary tumors with several studies achieving accuracies of 70–90% [121-123]. However, as biliary and pancreatic sphincterotomy and stent placement cannot be attained during EUS, those who require these therapeutic interventions must also undergo an ERCP.

### Staging and treatment

The TNM staging defines T1 stage tumor as limited to ampulla of Vater [50]. Tumors infiltrating duodenal wall are T2 and those infiltrating pancreas are T3. T2 tumors infiltrate into peripancreatic tissue or surrounding organs. Lymph node metastases around the superior mesenteric artery, the celiac trunk or pancreatic tail are considered as metastases. As in the former two biliary malignancies, the only potentially curative treatment for ampullary carcinoma is surgical resection. Entire tumor resection with negative margins is essential for cure. A Whipple’s operation is regarded as the standard approach for ampullary carcinoma; surgical outcomes have improved with time with rates of potentially curative resection increased from approximately 80 to over 90% [118,124].

An ampullectomy may be considered in those with early low-grade tumors. Some have proposed that local resection is a reasonable approach for small (<6 mm) well-differentiated tumors that do not penetrate through the ampullary musculature [125]. Despite the high rate of potentially curative resections, more than 50% of patients succumb to recurrent disease, suggesting the need for adjuvant therapy. Although there is no consensus regarding the optimal management of patients after resection, the benefit from post-operative chemoradiotherapy has been suggested by several studies [126].

Other malignant neoplasms of the bile ducts include lymphomas, leiomyosarcomas, carcinoid tumours and metastases in adults. Embryonal rhabdomyosarcoma can occur in children and is the second most common cause of jaundice in pediatric population. Readers are referred to literature elsewhere for more details on these rare neoplasms.

## Conclusion

Malignancies of the biliary tract are uncommon but associated with poor prognosis due to their late detection. CT, MRI with MRCP and EUS are most important in detection of early stage tumors and for pre-operative planning. MRI and MRCP is the single most useful modality for diagnosis of biliary malignancies. Surgical resection is the only curative treatment available although the 5-year survival is still poor compared to other tumors. Overall, imaging plays a critical role in the management of these malignancies and requires a combined evaluation with one or more modalities along with clinical features and histological grade of the malignancy.

## Abbreviations

CT: Computed tomography; MRI: Magnetic resonance imaging; MRCP: Magnetic resonance cholangiopancreatography; ERCP: Endoscopic retrograde cholangiopancreatography; EUS: Endoscopic ultrasound; PET: Positron emission tomography; CCA: Cholangiocarcinoma; HCC: Hepatocellular carcinoma; PSC: Primary sclerosing cholangitis; iCCA: Intrahepatic cholangiocarcinoma; pCCA: Perihilar cholangiocarcinoma; dCCA: Distal cholangiocarcinoma; AFP: Alpha-feto protein; AJCC/UICC: The American Joint Cancer Committee/Union for International Cancer Control; LSCGJ: Liver cancer study group of Japan; NCCJ: National Cancer center of Japan; EUSFNA: Endoscopic ultrasound guided fine needle aspiration; GbCA: Gallbladder carcinoma.

## Competing interests

The authors declare that they have no competing interests.

## Authors’ contributions

TPH carried out review of images, review of literature, drafted manuscript, review and finalization of manuscript. WTN carried out review of images, review of literature, drafted manuscript, review and finalization of manuscript. SKV carried out review of images, review of literature, drafted manuscript, review and finalization of manuscript. All authors read and approved the final manuscript.
